# Volatile Organic Compounds of the Plant Growth-Promoting Rhizobacteria JZ-GX1 Enhanced the Tolerance of *Robinia pseudoacacia* to Salt Stress

**DOI:** 10.3389/fpls.2021.753332

**Published:** 2021-10-14

**Authors:** Pu-Sheng Li, Wei-Liang Kong, Xiao-Qin Wu, Yu Zhang

**Affiliations:** ^1^Co-Innovation Center for Sustainable Forestry in Southern China, College of Forestry, Nanjing Forestry University, Nanjing, China; ^2^Jiangsu Key Laboratory for Prevention and Management of Invasive Species, Nanjing Forestry University, Nanjing, China

**Keywords:** *Rahnella aquatilis*, volatile organic compounds, salt stress tolerance, *Robinia pseudoacacia*, 2,3-butanediol

## Abstract

Salt stress is one of the major abiotic stresses that affects plant growth and development. The use of plant growth-promoting rhizobacteria to mitigcate salt stress damage in plants is an important way to promote crop growth under salt stress conditions. *Rahnella aquatilis* JZ-GX1 is a plant growth-promoting rhizobacterial strain, but it is not clear whether it can improve the salt tolerance of plants, and in particular, the role of volatile substances in plant salt tolerance is unknown. We investigated the effects of volatile organic compounds (VOCs) from JZ-GX1 on the growth performance, osmotic substances, ionic balance and antioxidant enzyme activities of acacia seedlings treated with 0 and 100mm NaCl and explored the VOCs associated with the JZ-GX1 strain. The results showed that compared to untreated seedlings, seedlings exposed to plant growth-promoting rhizobacterium JZ-GX1 via direct contact with plant roots under salt stress conditions exhibited increases in fresh weight, lateral root number and primary root length equal to approximately 155.1, 95.4, and 71.3%, respectively. *Robinia pseudoacacia* seedlings exposed to VOCs of the JZ-GX1 strain showed increases in biomass, soil and plant analyser development values and lateral root numbers equal to 132.1, 101.6, and 166.7%, respectively. Additionally, decreases in malondialdehyde, superoxide anion (O_2_^−^) and hydrogen peroxide (H_2_O_2_) contents and increases in proline contents and superoxide dismutase, peroxidase and glutathione reductase activities were observed in acacia leaves. Importantly, the sodium-potassium ratios in the roots, stems, and leaves of acacia exposed to VOCs of the JZ-GX1 strain were significantly lower than those in the control samples, and this change in ion homeostasis was consistent with the upregulated expression of the (Na^+^, K^+^)/H^+^ reverse cotransporter RpNHX1 in plant roots. Through GC-MS and creatine chromatography, we also found that 2,3-butanediol in the volatile gases of the JZ-GX1 strain was one of the important signaling substances for improving the salt tolerance of plants. The results showed that *R. aquatilis* JZ-GX1 can promote the growth and yield of *R. pseudoacacia* under normal and salt stress conditions. JZ-GX1 VOCs have good potential as protectants for improving the salt tolerance of plants, opening a window of opportunity for their application in salinized soils.

## Introduction

In natural environments, plants are usually exposed to various abiotic stresses, such as drought, salinity, and extreme temperatures. Salt stress is one of the major abiotic factors affecting plant growth (Thi et al., 2016). A number of methods have been developed to reduce the severe effects of salt stress on plants. At present, the improvement of salinized soil is mainly carried out through measures such as adjusting water resources, applying chemical fertilizers and growing salt-tolerant plants, but these methods have certain limitations, may require large energy inputs, are costly, are not sustainable for the environment and ecosystem (Bresler et al., 1982), and cannot fundamentally improve soil quality conditions or increase plant biomass (Li et al., 2019). In the last decade, the use of plant growth-promoting rhizobacteria (PGPR) has been shown to have significant advantages over other methods due to their beneficial effects on plant growth and stress tolerance. PGPR are economical, environmentally friendly, easy to detect and inexpensive (Batchelor et al., 1997). Therefore, PGPR have attracted widespread attention (Sheikh et al., 2016; Cheng et al., 2017; Baek et al., 2020).

It has been reported that PGPR can promote plant growth or increase plant stress resistance through the production of nonvolatile substances. For example, growth hormones, cytokinin, 1-aminocyclopropane-1-carboxylic acid deaminase, and iron are synthesized to promote the uptake of nutrients by roots (Loper and Schroth, 1981; Glick et al., 1999; Timmusk et al., 1999). In recent years, there has been increased interest in studying volatile organic compounds (VOCs) released by PGPR, and these VOCs play a significant role in promoting the growth and development of plants and resisting adverse environmental conditions (Kanchiswamy et al., 2015; Sharifah et al., 2019).

The effect of VOCs on plant growth was first identified by Ryu et al. (2003). *Bacillus subtilis* enhances vegetable growth, photosynthesis, iron uptake and disease resistance through the release of volatile chemicals (Zhang et al., 2007, 2008a, 2009). Under salt stress conditions, *Arabidopsis thaliana* treated with *B. subtilis* GB03 VOCs exhibited higher biomass and less sodium ion accumulation than control plants (Zhang et al., 2010), and this treatment promoted Na^+^ transfer from the ground to the roots by regulating the activity of the *A. thaliana* Na^+^ transporter protein HKT1 (Zhang et al., 2008b). Volatile gas production by interroot bacteria confers systemic tolerance to abiotic stresses by regulating the production of proline, antioxidants and hormones and reducing the accumulation of sodium ions in plants (Zhang et al., 2010; Liu and Zhang, 2015; Sharifi and Ryu, 2017). The main biologically active volatile compounds reported in microorganisms are 2,3 butanediol (Ryu et al., 2003; Farag et al., 2013), adipic acid, butyric acid (Farag et al., 2013), dimethylhexadecylamine (Batchelor et al., 1997; Nilsson et al., 2001), and tridecane (Lee et al., 2012). Of these compounds, 2,3 butanediol is the most widely reported (Ryu et al., 2003; Ji and Huang, 2011).

*Rahnella aquatilis* JZ-GX1 is a strain of plant-promoting bacteria that was isolated from the rhizosphere of *Pinus massoniana* in our previous study (Li and Wu, 2014). A previous study showed that JZ-GX1 significantly increased the germination rate, germination potential, fresh weight, primary root length and stem length of tomato seeds under salt stress, and it is a moderately salinophilic bacterium with good growth-promoting function (Li et al., 2021). However, it is not clear whether VOCs from this strain can promote plant growth under salt stress. Current studies on the interaction between plant growth-promoting rhizobacterial VOCs and plants have mostly focused on herbaceous plant species, with *A. thaliana* being the most commonly studied, and no reports on woody plant species have been published. Therefore, the present study was carried out using the woody plant species *Robinia pseudoacacia* as the study material with the aim of (i) understanding whether VOCs produced by *R. aquatilis* JZ-GX1 under salt stress conditions affect the tolerance of woody plants to salt stress; (ii) revealing how VOCs produced by *R. aquatilis* JZ-GX1 under salt stress conditions affect the tolerance of woody plants to salt stress; and (iii) exploring whether the VOC 2,3-butanediol produced by *R. aquatilis* JZ-GX1 under salt stress conditions is a signaling compound that affects plant salt tolerance. This study aimed to explore the mechanisms by which JZ-GX1 regulates plant salt stress responses to provide evidence that microorganisms producing 2,3-butanediol can enhance the salt tolerance of plants.

## Materials and Methods

### Test Strain and Culture Medium

*Rahnella aquatilis* JZ-GX1 is a plant growth-promoting bacterium that was isolated from the rhizosphere soil of 28-year-old *P. massoniana* in Nanning, Guangxi, and is currently stored in the Type Culture Preservation Center of China (CCTCC, No: M 2012439; Li and Wu, 2014).

LB solid medium was prepared as follows: 10g peptone, 5g yeast powder, 10g sodium chloride, 15–20g agar, pH 7.2. Additionally, 1/2-strength MS medium was purchased from Beijing Rong Shi De Biomedical Technology Co. Based on preliminary results (unpublished) and the study by Zhang et al. (2018) on soil salinity grading, the NaCl concentrations of the above media were set at 0 and 100mm. The activated test strain was placed in the media and shaken at 28°C and 200rpm until it reached the logarithmic phase of growth. The seed medium was prepared as follows: 10g glucose, 1g yeast powder, 2g peptone, 6g (NH_4_)_2_SO_4_, 10g KH_2_PO_4_, 0.5g NaCl, 0.5g MgSO_4_, pH 7.2. The fermentation medium was prepared as follows: 60g glucose, 20g peptone, 5g yeast powder, 0.5g KH_2_PO4, pH 7.0.

### Plant Material and Treatment

#### Plant Material Sources

The seeds of *R. pseudoacacia* used in the experiment were obtained from the Southern Forestry Seed Inspection Center of the State Forestry Administration, China.

#### Plant Material Treatments

Undamaged seeds that were uniform in size and fullness were surface-sterilized (soaked in 70% ethanol for 2min and 5% NaClO for 15min), washed thoroughly with sterile distilled water and air-dried to remove any surface water. The seeds were then sown in water agar Petri dishes and vernalized for 2days at 4°C in the dark. Then, the seeds were placed in a growth chamber followed by a light incubator and subsequently incubated at 28°C in the dark for germination prior to use.

Experimental setup with bacteria in direct contact with plant roots: Germinated seedlings were arranged in special square plastic plates (3 plants per plate) that contained 0mm or 100mm NaCl, and the seedlings were treated with a 1×10^7^cfu/ml bacterial suspension 5cm from the tip of the primary root. The experiment was replicated six times with 18 seedlings in each replicate.

Experimental setup with bacteria not in direct contact with plant roots: Germinated seedlings were transplanted into glass vials that contained two sections (first section: LB solid medium; second section: 1/2-strength MS solid medium). A control group (CK; 5μl sterilized PBS buffer) and a test group (JZ-GX1; 5μl JZ-GX1 suspension) were established. An experiment to verify whether 2,3-butanediol, which is one JZ-GX1 VOC, plays an important role in improving plant salt tolerance was performed by transplanting germinated seedlings into one side of a dichotomous dish (100×15mm) containing a central partition (plate I: LB solid medium; plate II: 1/2-strength MS solid medium). The treatments included (i) 5μl sterile water; (ii) 5μl JZ-GX1 suspension; and (ii) different concentrations of 2,3-butanediol.

The medium salt concentration was set to 100mm NaCl for all the cultures. The glass vials and plates were sealed with paraffin film and placed in a light incubator. The light incubator was set to a cycle of 12h of light and 12h of darkness at a temperature of 25±4°C and a relative humidity of 65±10% (Velázquez-Becerra et al., 2011). The experiments were independently performed eight times.

### Quantification of Biomass and Root Growth

The fresh weight of the seedlings was measured by an analytical balance after 10days of direct contact between *R. aquatilis* JZ-GX1 and plant roots. The length of the primary roots was measured with a straightedge, and the number of lateral roots was determined immediately after harvest. After 14days of exposure to *R. aquatilis* JZ-GX1 VOCs, the fresh weight of the seedlings was measured with an analytical balance, the length of the primary roots was measured with a straightedge, the number of lateral roots was determined immediately after harvesting, and plant soil and plant analyser development (SPAD) values were measured with a SPAD 502 Plus chlorophyll metre. The experiments were performed with three replicates with ten seedlings per replicate.

### Determination of MDA Content

Acacia leaves (0.5g) were weighed and exposed to JZ-GX1 VOCs for 14 d, cut and put into a mortar. Ten milliliters of 5% trichloroacetic acid and a small amount of quartz sand were added, the leaf pieces were ground into a homogenate and centrifuged at 3000rpm for 10min, and the supernatant was aspirated as the extraction solution. Then, 1ml of supernatant was transferred to a test tube, and 1ml of 0.67% TBA solution was added and shaken well (1ml of distilled water was added as a control). Then, the test tube was placed into boiling water for 30min, removed from the bath to cool, and centrifuged at 3000rpm for 10min. Finally, the supernatant was collected to measure its absorbance at 532, 450, and 600nm with TBA solution as a reference. C (μmol/L)=6.45×A532−A600–0.56×A450 (Li, 2000). The experiments were performed in triplicate with twenty seedlings per replicate.

### Determination of Proline Content

*Robinia pseudoacacia* leaves (0.1g) were weighed after 14d of exposure to JZ-GX1 VOCs. Three replicates were established for each treatment, and the plant proline content was measured after different treatments using a kit (Cominbio, Suzhou, China). The experiments were performed in triplicate with twenty seedlings per replicate.

### Ion Determination

The plant tissues were dried at 70°C after 14d of exposure to JZ-GX1 VOCs. The dried plant tissues were finely ground and passed through a lmm sieve, and lg of plant tissues was accurately weighed and placed in a 100ml triangular flask. Thirty milliliters of mixed acid (HNO_3_: HClO_4_: H_2_SO_4_; 8: 1: 1, V/V) was added, and a curved-neck funnel was placed at the mouth of the flask and left overnight. The following day, temperature-controlled decoction was performed in a fume hood with six adjustable electric furnaces. The solution in the triangular flask was maintained at a low boil until a large amount of brown nitrogen dioxide NO_2_ gas was released. When the brown gas disappeared, the furnace temperature was increased to dehydrate the silica until white smoke was produced. If the solution was still cloudy, 5ml an acid mixture was added, and heating was continued until the solution became clear and showed white smoke. After cooling, 20ml of deionized water was added, and the solution was filtered through filter paper into a 100ml volumetric flask. Then, the triangular flask and filter residue were washed with preheated 1% hydrochloric acid solution until there was no Fe^3+^ reaction. The volume was fixed with deionized water, and the solution was shaken well and measured (Lin et al., 2016). A sodium-potassium ion standard solution was prepared, and the sodium-potassium ion content in the standard and sample was determined with an FP6450 flame photometer. A standard curve was drawn to calculate the sodium-potassium concentrations of the samples. The experiments were performed with three replicates of twenty seedlings per replicate.

### Assays for Hydrogen Peroxide (H_2_O_2_), Superoxide Anion (O_2_^−^), Glutathione Reductase and Antioxidant Enzymes

Leaf samples (0.1g) were ground in liquid nitrogen and homogenized in an ice bath using 1ml of the extraction solution from the kit. Centrifuge, remove supernatant and place on ice for measurement.

The concentration of H_2_O_2_ was determined by measuring the absorbance of the titanium–hydroperoxide complex (Sui et al., 2007). The superoxide anion reacts with hydroxylamine hydrochloride to produce NO_2_^−^, NO_2_^−^ in the presence of p-aminobenzenesulfonic acid and naphthylamine to form a red azo compound with a characteristic absorption peak at 530nm. The O_2_^−^ generation was expressed as the content per gram of fresh leaf mass (Sui et al., 2007). The activity of superoxide dismutase (SOD) was determined with SOD assay kit and was presented as U/gFW. One unit of SOD activity is the amount of extract that gives 50% inhibition in reducing xanthine monitored at 560nm (Sunjeet et al., 2021). peroxidase (POD) activity was measured by using a POD assay kit based on the POD-catalyzed oxidation of a specific substrate by H_2_O_2_ witvh characteristic light absorption at 470nm and expressed as U/gFW. One unit of POD activity is the amount of enzyme, which causes the decomposition of 1μg substrate per minute in 1mg fresh sample at 37°C. Similarly, the activity of CAT was measured with a CAT assay kit and was presented as U/gFW. One unit of CAT activity is the amount of enzyme which causes the decomposition of 1μmol H_2_O_2_ per minute in 1mg fresh sample at 37°C (Sunjeet et al., 2021). The glutathione reductase (GSH) content was determined with a glutathione assay kit according to the DTNB [5, 5, − dithiobis (2-nitrobenzoic acid)] method. The absorbance was measured at 412nm, and GSH content was expressed as μmol/gFW.

The contents of H_2_O_2_, O_2_^−^, SOD, POD, CAT and GSH were measured with the corresponding assay kits (Cominbio, Suzhou, China) based on the manufacturer’s protocols. All experiments were performed with three replicates of ten seedlings per replicate.

### Detection of 2,3-Butanediol Production by *R. aquatilis* JZ-GX1

The colonies were inoculated into seed medium and incubated at 28°C and 200rpm until they reached the logarithmic growth phase. Then, the samples were inoculated into fermentation medium at 1% inoculum and incubated for 12h.

Indirect detection: Ethylene coumarin is a precursor substance of 2,3-butanediol. To detect ethylene coumarin by a colorimetric method, a certain amount of fermentation broth was collected and diluted in the following reaction system (10ml reaction system: 10% NaOH: 0.5% creatine: 5% 1-naphthol:deionized water=1:1:1:7), and the treatment setup included (i) an ethylene coumarin standard; (ii) JZ-GX1 fermentation broth; and (iii) H_2_O. After the reaction solution was shaken and mixed, 25μl the diluted fermentation solution was added immediately, the effect of the volume of the fermentation solution on the reaction system was ignored, and the colour change after the reaction was observed.

Direct detection: An appropriate amount of fermentation supernatant was collected, ethyl acetate was used as the extractant, and the extract and fermentation solution volume ratio was 1:1. GC-MS detection as performed with a DB-5MS chromatographic column (30m*0.25mm*0.25μm), and the column temperature cycle was as follows: held at 50°C for 1min, ramped up to 90°C at a rate of 6°C/min and held for 10min, and finally ramped up to 230°C at a rate of 20°C/min. The inlet temperature was 240°C, the injection volume was 1μl, the carrier gas flow rate was 1.0ml/min, and the carrier gas was high-purity helium. The TraceMS mass spectrometry conditions were as follows: EI+ bombardment source, full scan mode, scan mass range of 30–500amu, emission current of 200μA, and electron energy of 70eV. The mass spectrometry detection library was the NIST98 library (Yang, 2013). Our experiments were carried out independently three times.

### Quantitative Real-Time Polymerase Chain Reaction Analysis

Total plant RNA was isolated using an RNA kit (Beijing Zhuangmeng International Biogene Technology Co., Ltd.) according to the manufacturer’s instructions. cDNA samples were prepared using HiScript II Q Select RT SuperMix for qPCR (China). The expression of genes related to ion absorption, migration and compartmentalization was determined by qRT-PCR with an ABI 7500 (Applied Biosystems, United States), and atpD was used as an internal control (Jie et al., 2017). Two genes associated with Na^+^ ion uptake, movement and compartmentalization were identified in the study by Jie et al. (Table 1). The relative changes in gene expression were calculated by the 2−ΔΔCT method. The RT-PCR assay consisted of three independent experiments with three replicates of each experiment.

**Table 1:** Primers used in the RT-qPCR analysis.

Gene name	Primers (5′-3′)	Annealing temperature
actin	CCCAAATCATGTTTGAGACCTTCA	57
	CATAGATTGGCACAGTGTGACTCA	
RpSOS1	AAGGTTGGAATYTGSWTGTTA	60
	AATWGMRCTTTSCTSCCACAG	
RpNHX1	CTATGGAGAYATACATGCAGT	53
	AAGCTGCWCTRTTKACCTTCAA	

### Reproducibility of Results and Statistical Analysis

The data were subjected to analysis of variance and Duncan’s multiple comparison using SPSS 17.0 software, and the mean values plus standard errors and significance levels were calculated. Different letters indicate significant differences between control and JZ-GX1 inoculated plants in the control or treatment groups (*p*<0.05).

## Results

### *Rahnella aquatilis* JZ-GX1 Enhances Plant Tolerance to Salt Stress

**Figure 1:**
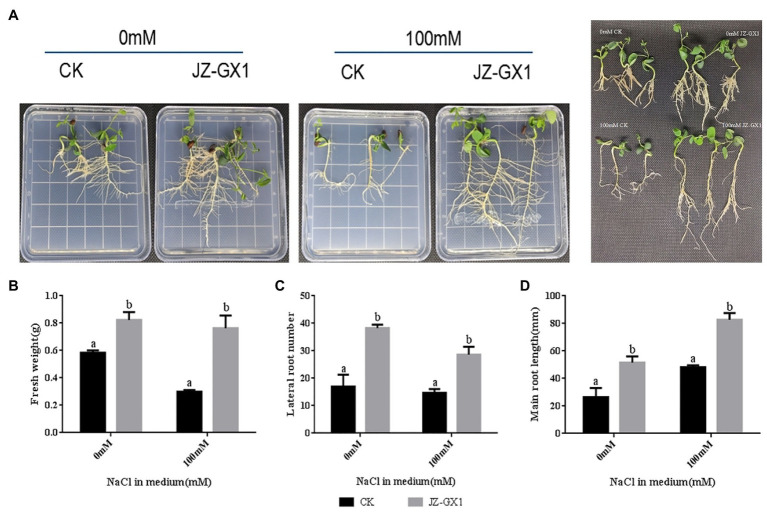
Effect of *Rahnella aquatilis* JZ-GX1 on the growth of *Robinia pseudoacacia*. **(A)** phenotype; **(B)** fresh weight; **(C)** lateral root number; **(D)** primary root length. Different letters indicate significant differences between treatments (*p*<0.05), and the same applies below.

To test whether the JZ-GX1 strain has a protective effect on plants under salt stress conditions when in direct contact with the plant root system, we performed growth assays using *R. pseudoacacia*. At day 10 of treatment, the plant size of seedlings cocultured with JZ-GX1 was slightly larger than that of the untreated control plants (Figure 1A). The fresh weight of seedlings treated with JZ-GX1 for 10days was measured (Figure 1B). The results showed that the fresh weight of JZ-GX-treated seedlings under non-salt stress conditions significantly increased by 41.1% compared with that of seedlings under control conditions, and the fresh weight of JZ-GX1-treated seedlings grown on salt media was 2.55 times heavier than that of untreated seedlings.

To investigate the effect of JZ-GX1 on plant root development under salt stress conditions, we determined the number of lateral roots and the length of the main root (Figures 1C,D). Under non-salt stress conditions, the JZ-GX1 treatment increased the number of lateral roots and the length of primary roots by approximately 123.5 and 94.6%, respectively, compared to the untreated control. Under salt stress (100mm), coculture with JZ-GX1 increased the number of lateral roots and the length of primary roots by approximately 95.4 and 71.3%, respectively.

**Figure 2:**
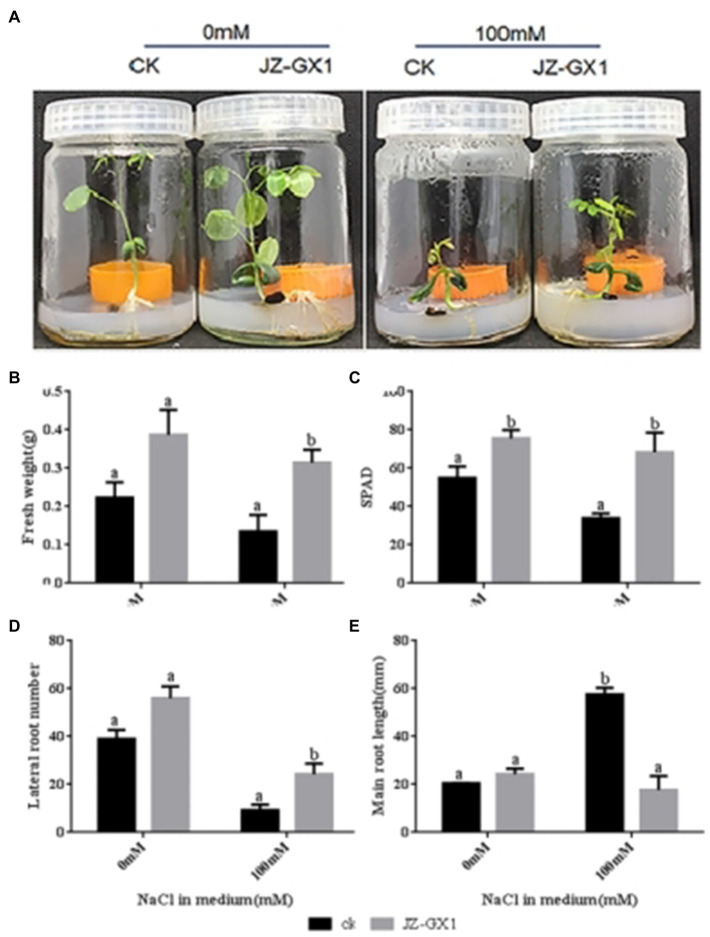
Effect of volatile gas from *R. aquatilis* JZ-GX1 on the growth of *R. pseudoacacia*. **(A)** phenotype; **(B)** fresh weight; **(C)** soil and plant analyser development value; **(D)** lateral root number; **(E)** primary root length.

Compared to acacia plants exposed to the water control, acacia plants exposed to JZ-GX1 volatiles showed robust growth when *R. aquatilis* JZ-GX1 did not directly contact the plant root system under either non-salt stress (0mm) or salt stress (100mm) conditions (Figure 2A). Fourteen days after treatment, plants exposed to JZ-GX1 volatiles clearly showed enhanced biomass on medium containing 0mm and 100mm NaCl (Figure 2B), and the chlorophyll content SPAD value increased by 45.4% (Figure 2C). This suggests that JZ-GX1 volatiles can promote photosynthesis through chlorophyll accumulation and enhance photosynthetic growth under salt stress conditions. JZ-GX1 volatiles were also found to reduce the primary root length but increase the number of lateral roots in acacia, which was 2.7 times higher under salt stress conditions than under the control water treatment conditions (Figures 2D,E). Therefore, JZ-GX1 can help plants alleviate salt stress by affecting the root structure and improving water uptake efficiency.

### Enhancement of the Osmoregulatory Ability of Acacia by *R. aquatilis* JZ-GX1

**Figure 3:**
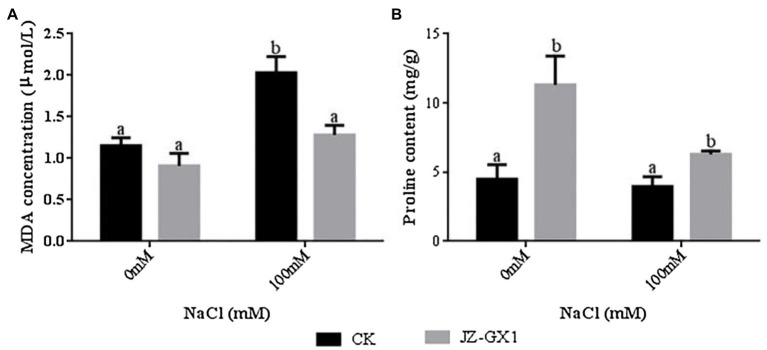
Effect of volatile gases of *R. aquatilis* JZ-GX1 on the malondialdehyde (MDA) content and proline osmoregulatory substance content in *R. pseudoacacia*. **(A)** MDA; **(B)** proline.

To study the changes in lipid peroxidation biomarkers after treatment with JZ-GX1 volatile compounds, we measured the level of malondialdehyde (MDA). The 100mm NaCl treatment increased the MDA content of the leaves of noninoculated and inoculated JZ-GX1 plants by 75.9 and 41.0%, respectively, compared to the 0mm NaCl treatment. Under normal and salt stress conditions, the MDA content of acacias cocultured with JZ-GX1 for 14days was reduced by approximately 27.1 and 58.5%, respectively, compared to that of untreated acacias. These results suggest that the plant growth-promoting rhizobacterium *R. aquatilis* JZ-GX1 reduced membrane oxidative damage in plants under salt stress. Proline is an amino acid that acts as an osmoprotectant and as a hydroxyl radical scavenger under various abiotic stress conditions (Hayat et al., 2012). In our study, *R. aquatilis* JZ-GX1 volatiles significantly increased the accumulation of proline in the plants (Figure 3).

### *Rahnella aquatilis* JZ-GX1 Regulates Na^+^ and K^+^ Homeostasis

**Figure 4:**
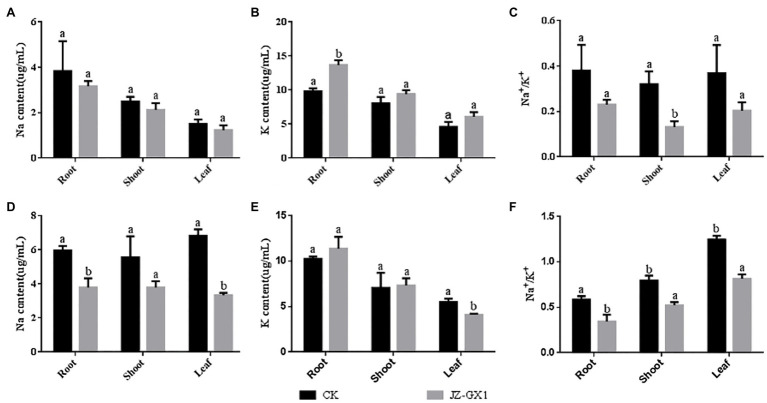
Effect of volatile gases produced by *R. aquatilis* JZ-GX1 on the distribution of Na^+^ and K^+^ contents in *R. pseudoacacia*. **(A)** 0mm Na^+^ content; **(B)** 0mmK^+^ content; **(C)** 0mm Na^+^/K^+^; **(D)** 100mm Na^+^ content; **(E)** 100mmK^+^ content; **(F)** 100mm Na^+^/K^+^.

Fourteen days after exposure, under non-salt stress, the Na^+^ accumulation in the roots, stems and leaves of *R. pseudoacacia* treated with volatile gases produced by *R. aquatilis* JZ-GX1 was not significantly different from that in the control samples, and the Na+distribution in the roots, stems and leaves showed the same trend, specifically following the order of leaves > roots > stems (Figure 4A). Under salt stress conditions, the total Na^+^ accumulation in acacias exposed to JZ-GX1 was 40.5% of the total accumulation in non-exposed plants (Figure 4D), indicating that the volatile gas produced by the JZ-GX1 strain reduced Na^+^ accumulation in acacia roots, stems, and leaves. The Na^+^ distribution under the CK treatment followed the order of leaves > roots > stems, but the Na^+^ distribution under the treatment with the volatile gas from JZ-GX1 followed the order of roots=stem > leaves. Additionally, the Na^+^ content in the aboveground and belowground parts was significantly lower after treatment with volatile gas from JZ-GX1 than after the CK treatment, indicating that the transport of Na^+^ from the roots to the aboveground parts was restricted under salt stress conditions.

In addition to reducing Na^+^ levels, under salt stress and non-salt stress conditions, JZ-GX1 exposure increased the total K^+^ content in *R. pseudoacacia* (Figures 4B,E). Specifically, with elevated NaCl (100mm) in the growth medium, an 11.1% increase in K^+^ accumulation in the roots and a 4.7% increase in the stems were observed in the presence of JZ-GX1 exposure, while K^+^ levels decreased by approximately 24.4% in the shoots (Figure 4E).

Exposure to volatile gas from *R. aquatilis* JZ-GX1 reduced the Na^+^/K^+^ level in *R. pseudoacacia* and leaves under both salt stress and non-salt stress conditions, helping plants resist salt stress (Figures 4C,F).

**Figure 5:**
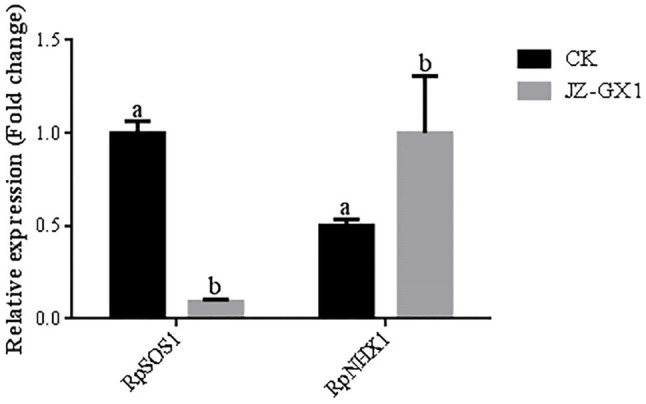
Effect of volatile gas produced by *R. aquatilis* JZ-GX1 on the expression of RpSOS1 and RpNHX1 in *R. pseudoacacia* root systems.

We further determined the genes associated with ion uptake, mobility and compartmentalization in acacia plants. In the root system, volatile gas produced by JZ-GX1 under salt stress conditions downregulated the expression of the RpSOS1 gene, which decreased by 9.0% compared to the control condition, while the expression of the RpNHX1 gene in inoculated plants was 99.2% higher than that in noninoculated plants after treatment with 100mm NaCl (Figure 5).

### Enhancement of the Antioxidant Capacity of Acacia by *R. aquatilis* JZ-GX1

**Figure 6:**
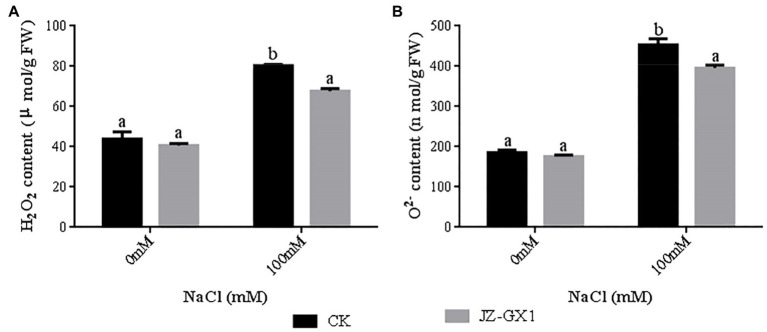
Effect of volatile gases produced by *R. aquatilis* JZ-GX1 on the H_2_O_2_ content of *R. pseudoacacia*. **(A)** H_2_O_2_ content; **(B)** O_2_^−^ content.

Salt stress significantly increased the H_2_O_2_ content (1.83-fold) and O_2_^−^ production rate (2.44-fold) in seedlings compared to non-salt stress conditions. The H_2_O_2_ content and O^2−^ production rate of JZ-GX1-inoculated seedlings were significantly reduced by 15.9 and 12.7%, respectively, under salt stress compared to those of the uninoculated control (Figure 6). This indicates that inoculation with JZ-GX1 can effectively reduce the accumulation of reactive oxygen species in seedlings under salt stress and alleviate oxidative stress.

**Figure 7:**
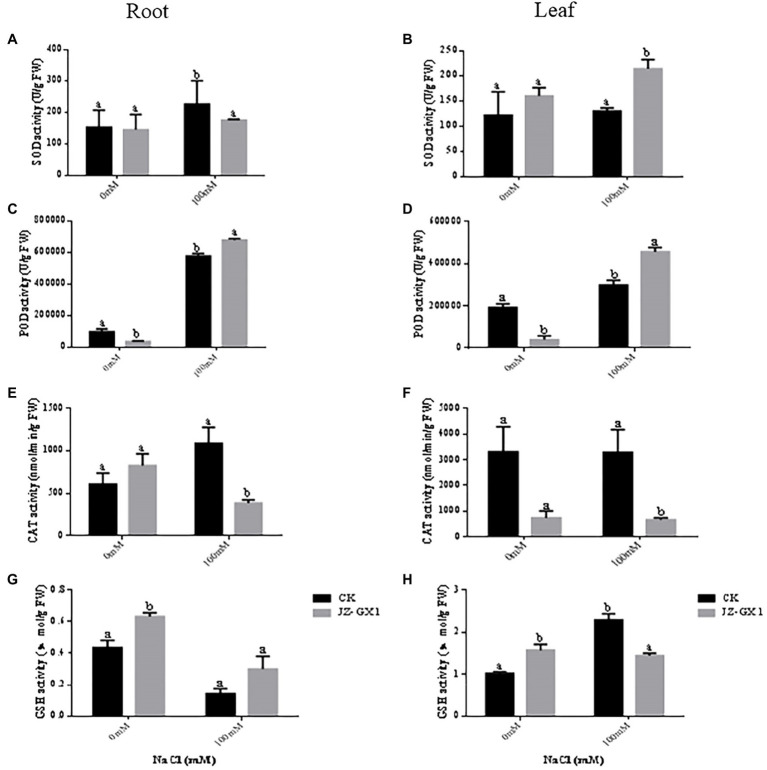
Effect of volatile gases produced by *R. aquatilis* JZ-GX1 on the superoxide dismutase (SOD), peroxidase (POD), CAT and glutathione reductase (GSH) activities in the leaves and roots of *R. pseudoacacia*. **(A)** Root SOD activity; **(B)** leaf SOD activity; **(C)** root POD activity; **(D)** leaf POD activity; **(E)** root CAT activity; **(F)** leaf CAT activity; **(G)** root GSH activity; **(H)** leaf GSH activity.

With increasing salt concentrations, the SOD activities of the leaves and roots of seedlings inoculated and not inoculated with JZ-GX1 increased (Figures 7A,B). In the roots, 100mm NaCl treatment increased the SOD activity of noninoculated and inoculated seedlings by 47.7 and 20.8%, respectively, compared to 0mm NaCl treatment. In the leaves, the SOD activity of seedlings inoculated with JZ-GX1 was increased by 6.6 and 33.8%, respectively, under 100mm NaCl treatment conditions compared to that under nonstressed conditions. Although in the root system, the leaf SOD activity of seedlings inoculated with JZ-GX1 was significantly lower than that of noninoculated seedlings under 100mm NaCl conditions, the leaf SOD activity of seedlings inoculated with JZ-GX1 was significantly higher than that of noninoculated plants under both 0 and 100mm NaCl conditions (31.5 and 65.0% higher than the latter, respectively).

In the roots, the POD activity of seedlings increased significantly with increasing salt concentrations. The POD activity of seedlings inoculated with JZ-GX1 decreased by 176.5% under nonstressed conditions and increased by 17.4% under stressed conditions compared with nonstressed conditions (Figure 7C). In leaves, the POD activity of seedlings without and with JZ-GX1 inoculation increased with increasing salt concentrations. The POD activity of seedlings without JZ-GX1 inoculation was 55.9% higher under the 100mm NaCl condition than under the nonstressed condition. The POD activity of seedlings inoculated with JZ-GX1 was 505.0% higher under the 100mm NaCl condition than under the nonstressed condition. Although the POD activity of inoculated seedlings was reduced under non-salt stress conditions compared with that of seedlings not inoculated with JZ-GX1, the POD activity of seedlings inoculated with JZ-GX1 was significantly increased by 52.7% under salt stress conditions (Figure 7D).

Under salt stress conditions, the CAT activities of the roots and leaves of JZ-GX1-inoculated seedlings were reduced, and only the CAT activities of the leaves were reduced in noninoculated plants (Figures 7E,F). Under non-salt stress conditions, inoculation with JZ-GX1 greatly increased the CAT activity of the seedling roots. In contrast, the CAT activity of the roots and leaves of seedlings inoculated with JZ-GX1 decreased significantly by 182.5 and 396.3%, respectively, under the 100mm NaCl treatment compared with those without inoculation.

In the roots, the GSH content was reduced by 206.3 and 108.8% in seedlings inoculated with JZ-GX1 and uninoculated seedlings, respectively, under 100mm NaCl treatment conditions compared to nonstressed conditions. The GSH content of the noninoculated plants did not change significantly with increasing salt concentrations (Figure 7G). The root GSH content of the plants inoculated with JZ-GX1 was significantly higher than that of the noninoculated plants after treatment with 0 and 100mm NaCl (44.9 and 112.5% higher than the latter, respectively). In the leaves, the GSH content of seedlings not inoculated with JZ-GX1 increased significantly with increasing salt concentrations. The GSH content of seedlings inoculated with JZ-GX1 decreased by 9.3% under 100mm NaCl treatment conditions compared with that under nonstressed conditions. The leaf GSH content of the seedlings inoculated with JZ-GX1 was significantly higher than that of the noninoculated seedlings under the 0mm NaCl condition (53.2% higher than the latter) but lower than that of noninoculated plants under the 100mm NaCl condition (Figure 7H).

### 2,3-Butanediol Is an Important Chemical Signal for the Enhancement of Salt Tolerance in *R. pseudoacacia* by *R. aquatilis* JZ-GX1

**Figure 8:**
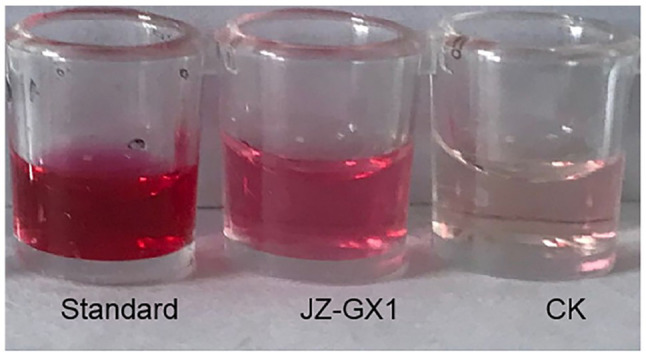
Determination of the 2,3-butanediol production ability of *R. aquatilis* JZ-GX1. (standard: 2,3-butanediol standard; JZ-GX1: JZ-GX1 fermentation solution; CK: H2O).

The results of the creatine chromogenic test showed that the supernatant of JZ-GX1 became pink after the addition of creatine chromogenic solution, while the blank control culture retained its original colour (Figure 8), indicating that strain JZ-GX1 could produce acetoin. Acetoin produces a precursor substance of 2,3-butanediol; therefore, these results indirectly indicated that the JZ-GX1 strain can produce 2,3-butanediol.

**Figure 9:**
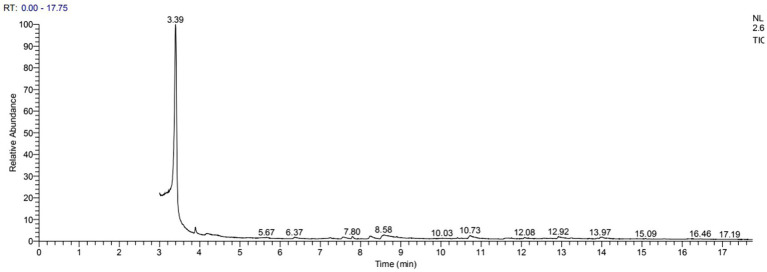
Spectrum of 2,3-butanediol identification by GC-MS in the fermentation broth of *R. aquatilis* JZ-GX1.

**Figure 10:**
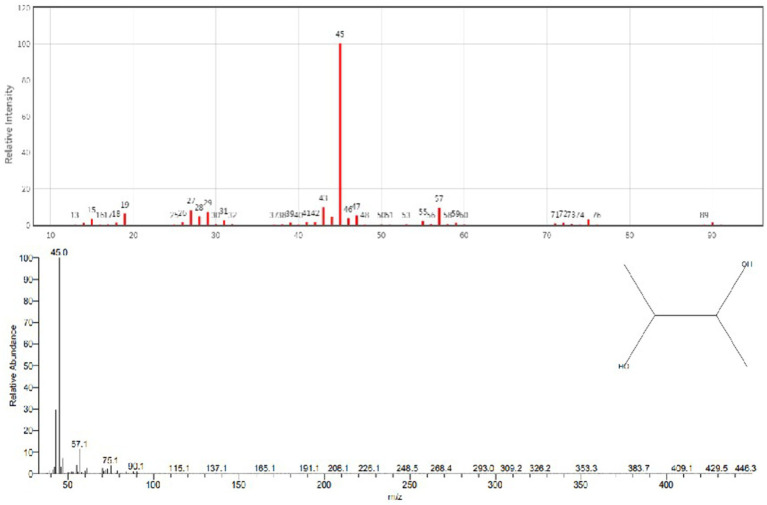
Gas chromatography–mass spectrometry analysis of the fermentation broth of *R. aquatilis* JZ-GX1.

GC–MS analysis showed that 2,3-butanediol could be produced by *R. aquatilis* JZ-GX1 by comparing the peak emergence time with the database (Figures 9, 10).

**Figure 11:**
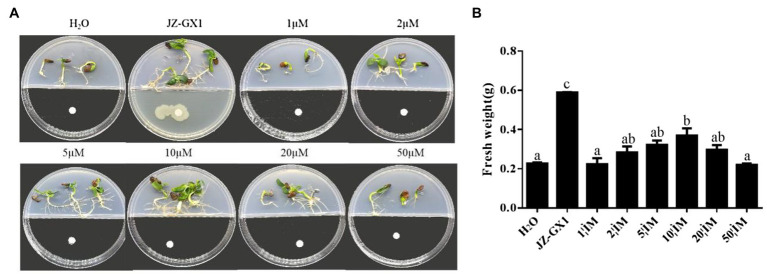
Effect of different concentrations of 2,3-butanediol on *R. pseudoacacia* under salt stress conditions. **(A)** Phenotype; **(B)** fresh weight.

To explore the contribution of 2,3-butanediol to the growth of *R. pseudoacacia* under salt stress conditions, different concentrations of 2,3-butanediol were used to test whether it could enhance plant growth and salt tolerance. The results showed that under salt stress conditions, the fresh weight of acacia increased significantly when incubated with 10μm 2,3-butanediol compared with the control. The biomass increased at 2,3-butanediol concentrations of 1, 2, 5, 20 and 50μm, but the difference was not significant. Plants exposed to the volatile gas of *R. aquatilis* JZ-GX1 showed the highest fresh weight (Figure 11).

### Summarizing the Main Mechanisms of Salt Tolerance in Plants Induced by *R. aquatilis* JZ-GX1 VOCs

**Figure 12:**
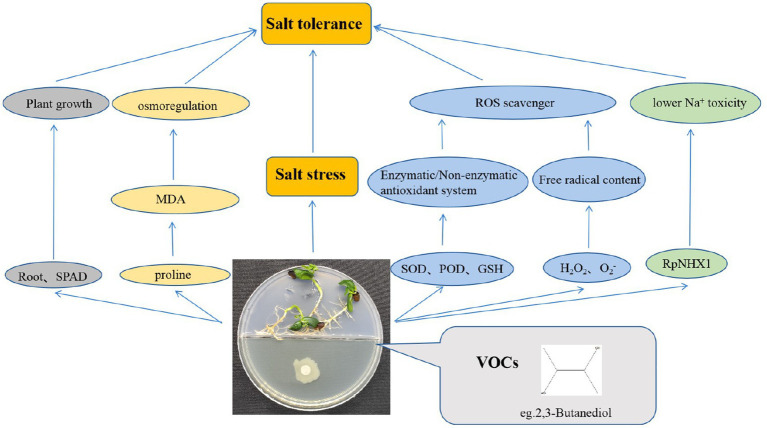
Main pathways of salt tolerance in plants induced by *R. aquatilis* JZ-GX1 volatile organic compounds.

With increasingly saline soils in the future (Otlewska et al., 2020), the effective use of saline soils is important. Exposure to JZ-GX1 VOCs modulates chlorophyll; alters root morphology; reduces MDA, superoxide anion and hydrogen peroxide contents; increases the proline osmoregulatory substance content; reduces intracellular sodium accumulation; and increases the antioxidant capacity to enhance stress tolerance under salt stress conditions in plants. Additionally, JZ-GX1 VOCs induce the differential expression of the (Na^+^, K^+^)/H^+^ reverse cotransporter RpNHX1. 2,3-Butanediol is also involved in VOC-induced plant resistance to salt stress (Figure 12).

## Discussion

Studies on plant growth-promoting rhizobacterial VOCs and plant resistance have included various strains of *Agrobacterium*, *Azospirillum*, *Bacillus*, *Pseudomonas* and *Rhizobium* (Ahmad et al., 2014). Among these strains, *Bacillus* and *Pseudomonas* strains are the most frequently studied (Ge et al., 2014). This study reports the effect of VOCs of *Rahnella* on plant salinity tolerance for the first time.

Some strains of PGPR play a crucial role in helping plants cope with adverse conditions, including salt stress (Zhang et al., 2010; Bresson et al., 2013). In this study, we showed that the plant growth-promoting rhizobacterium *R. aquatilis* JZ-GX1 improved plant growth and salt tolerance by promoting aboveground and root development when in contact with plant roots. VOCs emitted by JZ-GX1 enter the atmosphere as vapors and thus come into contact with plants due to their significantly high vapor pressure and low molecular weight (Dimkpa et al., 2009). Both plant biomass and SPAD values were significantly increased in plants grown on medium containing 100mm NaCl compared to the control medium. In addition, it is noteworthy that, in general, seedlings of the control-treated plants had only primary and secondary lateral roots, but seedlings treated with the JZ-GX1 strain produced tertiary and even quaternary lateral roots at the seedling stage, and the number of lateral roots was also 2.7 times higher than that under the control treatment. Patten and Glick (2002) reported that the promotion of lateral root growth is one of the main indicators of the beneficial effects of PGPR and that rapid root establishment through lateral root proliferation is beneficial to seedlings because it increases the ability of seedlings to anchor themselves to the substrate and obtain water and nutrients (Hommel et al., 2016). Better colonization of plant roots could also provide an advantage for bacteria by allowing them to obtain more root secretions and carbon sources (Yuan et al., 2020). In the natural environment, plant growth-promoting rhizobacterial VOCs diffusing through inter-root soil pores are mainly sensed by roots (Tahir et al., 2017). Thus, *R. aquatilis* JZ-GX1 appears to affect the root structure through changes in root development, which may enhance plant tolerance to salt stress. Since bacterial cells do not contact plant roots, VOCs produced by *R. aquatilis* JZ-GX1 under salt stress conditions play a role in improving the salt tolerance of plants.

The salt tolerance of plants depends largely on the amount of Na absorbed by the plant, the distribution of Na in the plant (Porcel et al., 2016), and the amount of Na^+^/K^+^ in the cytoplasm (Shabala and Cuin, 2008). Studies on wheat (*Triticum aestivum*; Talaat and Shawky, 2011) and *Tribulus terrestris* (*Medicago truncatula*; Garcia et al., 2017) revealed that Na^+^ accumulation in plants inoculated with arbuscular mycorrhizae was lower than that in noninoculated plants, and the proportion of Na allocated to aboveground parts was lower than that in noninoculated plants (Evelin et al., 2012; Porcel et al., 2016). The results of this experiment also showed that the volatile gas produced by *R. aquatilis* JZ-GX1 significantly increased K^+^ uptake by acacia under stress conditions. The reason for this may be, on the one hand, that the volatile gas produced by JZ-GX1 increases the number of phytometric roots and increases the contact area, thus effectively improving the ability of the prickly ash to absorb mineral nutrients from the culture substrate. On the other hand, K can be selectively absorbed by plants as an isotonic substance and transported to various plant organs and tissues, helping plants avoid the absorption of more Na^+^. The JZ-GX1 VOCs limit Na^+^ distribution in leaves and Na^+^ translocation to aboveground parts of seedlings, reducing plant Na^+^/K^+^ to maintain a stable ionic equilibrium. Blocking the translocation of Na^+^ from the root system to aboveground parts is an important strategy by which plants protect photosynthetic organs from Na^+^ toxicity (Zhu et al., 2016). A series of membrane transporter proteins responsible for K^+^ and Na^+^ uptake, movement and compartmentalization in plants plays a key role in reducing the excessive accumulation of Na elements (Wang et al., 2017). The cytoplasmic membrane Na^+^/H^+^ reverse cotransporter SOS1 and the vesicular membrane (Na^+^, K^+^)/H^+^ reverse cotransporter NHX1 are responsible for excreting Na^+^ from the cell or compartmentalizing it in vesicles, respectively (Qiu et al., 2002; Deinlein et al., 2014). These transporters represent two major mechanisms by which low Na^+^ levels are maintained in the cytosol (Deinlein et al., 2014). Because of the positive effects of JZ-GX1 in reducing plant Na^+^ contents, increasing K^+^ contents, and reducing leaf Na^+^/K^+^ contents, we further examined the expression of these 2 genes involved in K^+^ and Na^+^ uptake, transport, and compartmentalization. The results showed that in roots, JZ-GX1 VOCs upregulated the expression of RpNHX1 under stress conditions, which was in agreement with the results of (Porcel et al., 2016) obtained in rice (*O. sativa*) plants. Upregulation of RpNHX1 expression in roots promotes Na^+^ efflux from the intracellular space to the soil or plastid space, and the compartmentalization of Na^+^ in vesicles facilitates osmoregulation by cells. In fact, OsNHX1-4, which is located in the vesicles in rice, functions as a reverse vesicular (Na^+^, K^+^)/H^+^ cotransporter. Therefore, further studies on the expression of other genes encoding NHX proteins are needed to more clearly elucidate the contribution of these genes to Na^+^ compartmentalization in different regions of inoculated plants. In addition, JZ-GX1 VOC treatment reduced the expression of RpSOS1 in the root system, which differed from the results of Estrada et al. obtained in rice. Estrada et al. (2013) studied maize (*Zea mays*) inoculated with different arbuscular mycorrhizal fungi (AMF), including *Rhizophagus irregularis*, *Claroideoglomus etunicatum* and *Septoglomus constrictum*, and found that AMF could better regulate the expression of ZmSOS1 in the root systems of host plants. We speculate that the use of different plants and strains in the experiment is the reason for this inconsistent result.

Salt stress induces excessive production of ROS in plant cells, leading to membrane lipid peroxidation and disrupting the cell membrane integrity (Gill and Tuteja, 2010). In particular, very low concentrations of the superoxide anion hydrogen peroxide are required by plants for intracellular signal transduction processes. However, hydrogen peroxide overproduction inhibits the growth of plant roots (Cerny et al., 2018). In this study, the H_2_O_2_ content and O_2_^−^ production rate were higher in acacia seedlings under salt stress conditions, indicating that the cells were under oxidative stress and that the cell membranes were damaged. In contrast, the H_2_O_2_ and O_2_^−^ levels of inoculated seedlings were consistently lower than those of uninoculated seedlings under salt stress conditions. This is in agreement with Alexander et al. (2020), who showed that the application of *Stenotrophomonas maltophilia* BJ01 led to a decrease in the reactive oxygen levels in peanuts (*Arachis hypogaea*) under salt stress conditions. Enzymatic antioxidant systems (e.g., POD, SOD, and CAT) and nonenzymatic components (e.g., GSH and proline) play key roles in the induction, elimination, detoxification or neutralization of toxic levels of ROS (Liebthal et al., 2018). SOD is the first line of defense against damage caused by ROS (Apel and Hirt, 2004). The results of the experiment showed that the SOD activity in the leaves of the inoculated plants was significantly higher than that of the noninoculated plants at a concentration of 100mm NaCl, reflecting that the ability of the inoculated plants to scavenge O_2_^−^ under salt stress conditions was higher than that of the noninoculated plants. After SOD catalyses O_2_^−^ via a disproportionation reaction to H_2_O_2_ and O_2_^−^, other ROS-scavenging enzymes are required to quickly and efficiently remove H_2_O_2_ (Li et al., 2020). POD, CAT and GSH can all play a role in this process (Li et al., 2012). Inoculation with JZ-GX1 under salt stress conditions significantly increased the activity of POD in plant leaves and the content of GSH in the root system. The effect on CAT activity was not consistent with our expectations, as JZ-GX1 decreased CAT activity under stress conditions. However, overall, plants inoculated with JZ-GX1 had a stronger ability to scavenge reactive oxygen species than did noninoculated plants, and therefore, their MDA content was lower than that of noninoculated plants, although their CAT activity was also lower. Furthermore, the improvement of salt tolerance in plants by inoculation with JZ-GX1 did not cause an increase in the activity of all antioxidant enzymes. Thus, our results suggest that VOCs from the plant growth-promoting rhizosphere bacterium *R. aquatilis* JZ-GX1 can jointly regulate the antioxidant capacity of plants to prevent the oxidative damage caused by reactive oxygen species through both enzymatic and nonenzymatic systems. In addition, we found that volatile 2,3-butanediol helps protect plants from abiotic stresses and is the “chemical language” used by *R. aquatilis* JZ-GX1 VOCs to interact with their plant partners, which is consistent with results Sharifi and Ryu (2018). Volatile 2,3-butanediol helps protect plants from abiotic stresses. Treatment with the *Pseudomonas aeruginosa* O6 mutant, which is unable to synthesize 2,3-butanediol, did not improve drought stress tolerance in Arabidopsis compared with the wild-type strain. Notably, the effect of JZ-GX1 VOCs was better than the optimal concentration of pure 2,3-butanediol (10μm), suggesting the presence of other unknown gases with biological activity inducing plant resistance. The production and accumulation of substances has an impact on the signaling pathways induced by high-salt stress conditions and helps mitigate the effects of high salt concentrations (Faisal and John, 2020). Thus, 2,3-butanediol can be used as a promising tool for improving plant stress resistance.

## Conclusion

In conclusion, we observed a significant plant growth-promoting effect of *R. aquatilis* JZ-GX1 VOCs on *R. pseudoacacia* seedlings under salt stress conditions. *R. aquatilis* JZ-GX1 can help counteract stress through several mechanisms, mainly those involving the plant root conformation, the sue of osmoregulatory substances, reactive oxygen species scavenging and the reduction of sodium toxicity, as well as volatile 2,3-butanediol, which helps protect plants from abiotic stress. Our findings provide an effective and sustainable approach for the development of new microbial resources and the bioprotection of plants under salt stress conditions.

## Data Availability Statement

The original contributions presented in the study are included in the article/supplementary materials, further inquiries can be directed to the corresponding author/s.

## Author Contributions

P-SL completed the data analysis and the first draft of the paper. P-SL and W-LK completed the experimental research. YZ participated in the experimental results analysis. X-QW directed the experimental design, data analysis, paper writing, and revision. All authors read and agreed on the final version of the text.

## Funding

This work was supported by the National Key Research and Development Program of China (2017YFD0600104) and the Priority Academic Program Development of the Jiangsu Higher Education Institutions (PAPD).

## Conflict of Interest

The authors declare that the research was conducted in the absence of any commercial or financial relationships that could be construed as a potential conflict of interest.

## Publisher’s Note

All claims expressed in this article are solely those of the authors and do not necessarily represent those of their affiliated organizations, or those of the publisher, the editors and the reviewers. Any product that may be evaluated in this article, or claim that may be made by its manufacturer, is not guaranteed or endorsed by the publisher.
